# Evaluation of inpatient antibiotic prescribing patterns using WHO indicators in Northern Ethiopia: a prospective cross-sectional study identifying stewardship gaps

**DOI:** 10.1186/s13104-026-07852-0

**Published:** 2026-05-04

**Authors:** Haftom Yirga Tsegay, Berhane Yohannes Hailu, Gebrehiwot Gebremedhin Tafere, Filmon Beyenne Demoz, Werkey Araya Tekleargis, Kald Beshir Tuem

**Affiliations:** 1 Department of Veterinary Basics and Diagnostic Sciences, College of Veterinary Sciences, Mekelle University, Mekelle, Ethiopia; 2 Department of Pharmacology, School of Pharmacy, College of Health Sciences, Mekelle University, Mekelle, Ethiopia; 3 Department of Clinical Pharmacy, School of Pharmacy, College of Health Sciences, Mekelle University, Mekelle, Ethiopia

**Keywords:** Antibiotics, Antimicrobial stewardship, Drug utilization, Inpatients, Prescription pattern, World Health Organization

## Abstract

**Objective:**

This study aimed to evaluate inpatient antibiotic prescribing patterns and identify antimicrobial stewardship (AMS) gaps using World Health Organization (WHO) antibiotic use indicators at Ayder Comprehensive Specialized Hospital (ACSH), a major tertiary hospital in Northern Ethiopia.

**Results:**

Among 1,684 admitted patients, 865 (51.4%) received at least one antibiotic. A total of 1,491 antibiotics were prescribed, with a mean of 1.7 ± 0.7 antibiotics per patient and an average treatment duration of 5.9 ± 3.9 days. Nearly all antibiotics were prescribed by generic name (98.9%) and from the national Essential Medicines List (100%). Injectable formulations accounted for 90.9% of prescriptions. Ceftriaxone (41.0%), metronidazole (21.9%), and vancomycin (9.4%) were the most commonly used antibiotics. Watch-class antibiotics constituted 64.1% of prescriptions. More than half of antibiotic use was for therapeutic purposes (54.3%), of which 95.5% was empirical. Culture testing was performed in only 2.4% of patients, and antimicrobial susceptibility testing in 0.3%. Stock-outs affected 24.3% of antibiotics, with a mean duration of 4.8 days per month. Longer hospital stays, comorbidities, and severe clinical conditions, including sepsis and pneumonia, were associated with increased antibiotic use.

## Introduction

Antibiotics are crucial in the treatment of bacterial infections and have significantly contributed to increasing human life expectancy by saving millions of lives [1]. However, their effectiveness is increasingly threatened by the global rise of antimicrobial resistance (AMR) [2]. AMR is a major global health challenge that not only threatens clinical outcomes but also imposes significant economic burdens by prolonging hospital stays, increasing the need for costly treatments, and reducing the productivity of patients and caregivers [3].

The global burden of AMR is alarming. Globally, an estimated 1.27 million deaths were directly attributed to bacterial AMR in 2019, while 4.95 million deaths were associated with resistant infections [4]. These numbers continue to grow, by 2021, over 4.7 million deaths were linked to AMR, and projections indicate that by 2050, the annual toll could reach 8 million deaths [5]. The World Health Organization (WHO) continues to report alarming levels of resistance to commonly used antibiotics [6], underscoring the urgency of optimizing antibiotic use to preserve their effectiveness [7].

Inappropriate or irrational antibiotic use is recognized as the main driver of AMR, particularly in low- and middle-income countries (LMICs) where prescribing is often empirical, diagnostic support is limited, and antimicrobial stewardship (AMS) programs are poorly established [8, 9]. Contributing factors include inadequate infection prevention and control, unrestricted antibiotic access, poor prescriber adherence to treatment guidelines, and the lack of microbiological testing capacity [10].

To address this, the WHO Global Action Plan on AMR (2015) emphasized optimizing antimicrobial use as one of its core objectives [11]. The WHO also developed standardized antibiotic use indicators to evaluate and guide rational prescribing practices in healthcare facilities [12]. These indicators help identify stewardship gaps, benchmark prescribing behavior, and inform policy and intervention design [13, 14].

In Ethiopia, although full inpatient antibiotic prescribing evaluations are limited, the available evidence shows varied but concerning trends. For example, cross-sectional and observational studies in Debre-Tabor, Axum, Hawassa, and Fitche hospitals revealed high rates of antibiotic prescription, empiric prescribing, prolonged duration of antibiotic use, and frequent reliance on ceftriaxone [13–16]. Studies conducted at Ayder Comprehensive Specialized Hospital (ACSH), the second major hospital in the country, were limited to one or two departments/wards but still revealed high usage of cephalosporins and metronidazole. More than half of the admitted patients received multiple antibiotics, while culture and sensitivity testing were rarely performed [17, 18]. These findings collectively reveal persistent stewardship gaps, reflecting trends of high empiric prescribing, heavy reliance on a few antibiotic classes, limited microbiological support, and highlight the urgent need for systematic evaluation of antibiotic prescribing patterns using standardized methods.

Therefore, this study aimed to evaluate inpatient antibiotic prescribing and stewardship gaps using WHO antibiotic use indicators at ACSH, Northern Ethiopia. Identifying the key areas of irrational use and institutional bottlenecks is critical to guide AMS interventions, improve rational antibiotic use, and contribute to national and global efforts to combat AMR. This study can also help policymakers improve institutional and national antibiotic monitoring and regulation.

## Methods

### Study design, setting, and period

A prospective cross-sectional study was conducted among hospitalized patients receiving antibiotics in the surgical, medical, pediatric, and gynecology/obstetrics (Gyn/Obs) wards of Ayder Comprehensive Specialized Hospital (ACSH), a tertiary referral hospital in Northern Ethiopia with functional 500 inpatient beds.

Documents in the hospital indicated the hospital has basic structures in place to support antibiotic use, including the national Standard Treatment Guideline (STG), a Drug and Therapeutics Committee (DTC), and the national formulary list. However, no clinical guidelines were available for the management of infectious diseases.

Moreover, during the study period, the hospital microbiology laboratory provided limited routine culture services, including blood and urine cultures. Other specimen types (such as stool and wound swabs) were processed intermittently depending on severity of cases. Antimicrobial susceptibility testing was available for selected bacterial isolates; however, it was not performed for all cultured specimens due to resource and reagent constraints. The study was conducted from 16 June to 18 September 2024.

### Study population and eligibility

All patients admitted to the selected study wards during the study period and prescribed at least one systemic antibiotic were included, while those receiving long-term programmed therapy, such as antituberculosis or antiretroviral drugs, were excluded.

### Sample size and sampling

The sample size was calculated using a single population proportion formula, assuming a 50% prevalence of antibiotic use, a 95% confidence level, and a 3.5% margin of error, consistent with previous studies [13, 14]. After adding a 10% contingency, the final sample size was 865. Of 1,684 admitted patients, those who received at least one antibiotic were recruited using a quota-based systematic sampling technique.

### Ethical approval and consent

Ethical approval for this study was obtained from the Institutional Review Board (IRB) of Mekelle University (reference number: MU-IRB 2210/2024). The study was conducted in accordance with the Declaration of Helsinki. As the study involved prospective review of inpatient medical records without collection of personal identifiers or direct patient contact, the Mekelle University Institutional Review Board waived the requirement for informed consent. Permission to access patient records was obtained from Ayder Comprehensive Specialized Hospital administration prior to data collection.

### Data collection

Data were collected through medical chart review using a structured abstraction format adapted from the WHO antibiotic use indicators [12] and similar studies [13, 14]. Data on antibiotic availability were obtained from hospital pharmacy stock records. A stock-out was considered as the unavailability of a specific antibiotic in the hospital pharmacy at any time during the study period.

### Data quality assurance

The data collection tool was pretested on 5% of the sample, which was excluded from the final analysis. Eight trained data collectors conducted the chart reviews under close supervision, and record completeness was checked daily.

### Data analysis

Data were entered into EpiData version 4.6 and analyzed using SPSS version 25 and STATA version 14.2. Descriptive statistics were used to summarize patient characteristics and WHO antibiotic use indicators. Antibiotic stock-out results were expressed as the proportion of antibiotic items that experienced at least one stock-out during the study period. Factors associated with the distinct number of antibiotics prescribed were assessed using a generalized Poisson regression (GPR) model. Antibiotics were counted as distinct agents based on their generic names. Switching from one antibiotic to another was counted as separate antibiotic if a different agent was prescribed, whereas continuation of the same antibiotic was counted once per patient. Results were reported as incidence rate ratios (IRRs) with 95% confidence intervals (CIs). Model fit was evaluated using likelihood ratio tests, Akaike and Bayesian information criteria, and dispersion tests. Multicollinearity was assessed using variance inflation factor and tolerance. Statistical significance was set at *p* < 0.05.

## Results

### Sociodemographic characteristics

Most patients were male and from rural areas (Table 1).

**Table 1:** Sociodemographic characteristics of patients in the study wards, ACSH

Variable	Category	Study Wards, *n* (%)				Total *n* (%)
		Surgical	Medical	Paediatric	Gyn/Obs	
Sex	Male	204 (46.4)	117 (26.6)	119 (27.0)	0 (0.0)	440 (50.9)
	Female	70 (16.5)	116 (27.3)	57 (13.4)	182 (42.8)	425 (49.1)
Age (years)	Mean ± SD	36 ± 15	47 ± 20	6.3 ± 5.7	26 ± 8.1	-
Residence	Urban	101 (34.6)	46 (15.7)	84 (28.8)	61 (20.9)	292 (33.8)
	Rural	173 (30.2)	187 (32.6)	92 (16.1)	121 (21.1)	573 (66.2)

### Common conditions for which antibiotics were prescribed

Antibiotics were prescribed for infectious diseases (75.5%) and for surgical prophylaxis purposes (24.5%). They were most frequently prescribed for acute appendicitis (7.0%), pneumonia (6.7%), pyelonephritis (5.3%), and sepsis (4.9%) (Table 2). A majority of patients (62.4%) had one or more comorbidities, likely influencing the use of multi-drug regimens and prolonged therapy.

**Table 2:** Common conditions for which antibiotics prescribed in the study wards, ACSH

Conditions	Study Wards, *n* (%)				Total, *n* (%)
	Surgical	Medical	Pediatric	GYN/OBS	
Pneumonia	1 (0.1)	50 (5.8)	7 (0.8)	0 (0.0)	58 (6.7)
Traumatic injuries	77 (8.9)	0 (0.0)	33 (3.8)	0 (0.0)	110 (12.7)
Parapneumonic Effusion/Empyema	9 (1.0)	0 (0.0)	4 (0.5)	0 (0.0)	13 (1.5)
Sepsis	0 (0.0)	12 (1.4)	15 (1.7)	15 (1.7)	42 (4.9)
Abscess	13 (1.5)	2 (0.2)	2 (0.2)	1 (0.1)	18 (2.0)
Acute appendicitis	43 (5.0)	0 (0.0)	17 (2.0)	0 (0.0)	60 (7.0)
Meningitis	0 (0.0)	8 (0.9)	14 (1.6)	0 (0.0)	22 (2.5)
Pyelonephritis	8 (0.9)	25 (2.9)	11 (1.3)	2 (0.2)	46 (5.3)
Peritonitis	16 (1.9)	0 (0.0)	0 (0.0)	1 (0.1)	17 (2.0)
Hemothorax	13 (1.5)	0 (0.0)	2 (0.2)	0 (0.0)	15 (1.7)
Pneumothorax	11 (1.3)	0 (0.0)	0 (0.0)	0 (0.0)	11 (1.3)
Cholelithiasis	15 (1.7)	0 (0.0)	0 (0.0)	0 (0.0)	15 (1.7)
Opportunistic infections	2 (0.2)	16 (1.9)	3 (0.3)	4 (0.5)	25 (2.9)
Surgical site infections	0 (0.0)	0 (0.0)	0 (0.0)	4 (0.5)	4 (0.5)
Urinary tract infection	0 (0.0)	5 (0.6)	2 (0.2)	1 (0.1)	8 (0.9)
Stroke	0 (0.0)	26 (3.0)	0 (0.0)	0 (0.0)	26 (3.0)
Incomplete abortion	0 (0.0)	0 (0.0)	0 (0.0)	16 (1.9)	16 (1.9)
Surgical prophylaxis	55 (6.4)	0 (0.0)	26 (3.0)	131 (15.1)	212 (24.5)
Other medical conditions*	11 (1.3)	88 (10.2)	40 (4.6)	4 (0.5)	143 (16.5)
Unknown conditions	0 (0.0)	1 (0.1)	0 (0.0)	3 (0.3)	4 (0.5)
All conditions					865 (100)

*Includes anemia, chronic heart failure, diabetes mellitus, hypertension, severe acute malnutrition, pancytopenia, deep vein thrombosis, chronic liver disease, asthma, poisoning, myocardial infarction, and peptic ulcer disease

### WHO antibiotic use indicators

#### Prescribing indicators

A total of 1,491 antibiotics were prescribed for 865 inpatients, yielding an average of 1.7 ± 0.7 antibiotics per patient (Table 3).

**Table 3:** Prescribing indicators of antibiotics among patients in the study wards, ACSH

Indicator	Study Wards, *n* (%)									Total, *n* (%), mean ± SD	
	Surgical		Medical			Pediatric	GYN/OBS				
Number of antibiotic prescriptions*	432		468			338	253			1491	
The average number of antibiotics prescribed per patient	1.6		2			1.9	1.4			1.7 ± 0.7	
Antibiotics prescribed from national EML	432 (100)		468 (100)			338 (100)	253 (100)			1491 (100)	
Antibiotics prescribed by generic name	432 (100)		460 (98.3)			329 (97.3)	253 (100)			1474 (98.9)	
Antibiotics prescribed in injection form	431 (99.8)		382 (81.6)			319 (94.8)	223 (88.1)			1355 (90.9)	
Average duration of days antibiotic prescribed in the hospital stay	5.9		6.4			7.3	2.4			5.9 ± 3.9	
Number of admitted patients**	443		506			297	438			1684	
Patients admitted with at least one antibiotic	274 (62.0)		233 (46.0)			176 (59.3)	182 (41.6)			865 (51.4)	
Patients who received antibiotics for therapeutic purposes***	56 (20.4)		221 (94.8)			140 (79.5)	53 (29.1)			470 (54.3)	
Patients who received antibiotics for prophylaxis	218 (79.6)		11 (4.7)			36 (20.4)	126 (69.2)			391 (45.2)	
Patients who received antibiotics for an unknown purpose	0 (0.0)		1 (0.4)			0 (0.0)	3 (1.6)			4 (0.5)	
Patients who received antibiotics for empiric therapy	54 (96.4)		217 (98.2)			126 (90.0)	52 (98.1)			449 (95.5)	

*: Denominator for antibiotic prescription related calculations at ward level; **: Denominator for patient related calculations at ward level; ***: Denominator for empiric therapy calculations

#### Hospital and patient care indicators

Of the antibiotics included in the study, 24.3% experienced at least one stock-out during the study period, with stock-outs lasting an average of 4.8 days per month. The most frequently stocked-out antibiotics were amoxicillin-clavulanic acid, ceftriaxone and cefazolin. Microbiological support was extremely limited: only 21 patients (2.4%) had cultures performed, and of these, just 3 patients (14.3%) underwent antibiotic sensitivity testing, corresponding to 0.3% of all inpatients.

#### Distribution of prescribed antibiotics

The most frequently prescribed antibiotics were ceftriaxone (41%), metronidazole (21.9%), and vancomycin (9.4%) (Table 4).

**Table 4:** Antibiotics prescribed for patients in the study wards, ACSH

Antibiotics	Study Wards, *n* (%)				Total, *n* (%)
	Surgical	Medical	Pediatric	Gyn/Obs	
Ceftriaxone	256 (59.3)	158 (33.8)	134 (39.6)	64 (25.3)	612 (41.0)
Azithromycin	0 (0)	55 (11.7)	9 (2.7)	2 (0.8)	66 (4.4)
Metronidazole	146 (33.8)	74 (15.8)	59 (17.4)	48 (19.0)	327 (21.9)
Vancomycin	14 (3.2)	80 (17.1)	45 (13.3)	1 (0.4)	140 (9.4)
Gentamicin	0 (0)	4 (0.8)	8 (2.4)	0 (0)	12 (0.8)
Ampicillin	0 (0)	6 (1.3)	31 (9.2)	0 (0)	37 (2.5)
Doxycycline	0 (0)	2 (0.4)	0 (0)	21 (8.3)	23 (1.5)
Cefalexin	1 (0.2)	6 (1.3)	0 (0)	2 (0.8)	9 (0.6)
Meropenem	3 (0.7)	10 (2.1)	1 (0.3)	1 (0.4)	15 (1.0)
Ciprofloxacin	1 (0.2)	18 (3.8)	9 (2.7)	0 (0)	28 (1.9)
Amoxicillin	0 (0)	2(0.4)	7 (2.1)	0 (0)	9 (0.6)
Cefepime	0 (0)	4 (0.8)	0 (0)	0 (0)	4 (0.3)
Ceftazidime	11 (2.5)	43 (9.2)	31 (9.2)	3 (1.2)	88 (5.9)
Trimethoprim-sulfamethoxazole	0 (0)	2 (0.4)	4 (1.2)	0 (0)	6 (0.4)
Amoxicillin-clavulanic acid	0 (0)	4 (0.8)	0 (0)	0 (0)	4 (0.3)
Cefazolin	0 (0)	0 (0)	0 (0)	109 (43.1)	109 (7.3)
Cefixime	0 (0)	0 (0)	0 (0)	2 (0.8)	2 (0.1)
Total, n (%)	432 (29.0)	468 (31.4)	338 (22.7)	253 (17.0)	1491 (100)

**Fig. 1:**
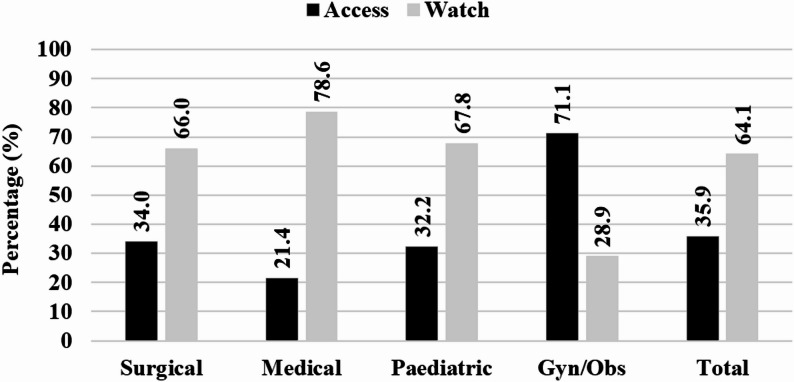
Proportion of Watch and Access of antibiotics, ACSH

Of all the prescribed antibiotics, 64.1% of them belonged to the Watch category and 35.9% to the Access category, as shown in Fig. 1.

**Fig. 2:**
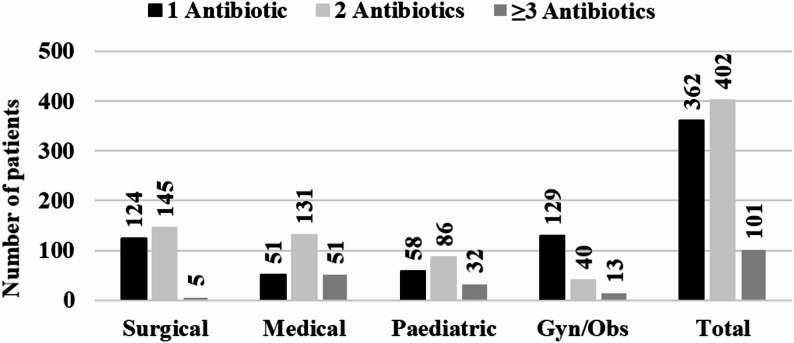
Number of antibiotics prescribed per patient in the study wards, ACSH

Combination therapy was common: 503 patients (58.2%) received multi-drug regimens, of which 402 (79.9%) received two-drug combinations, and 101 (20.1%) received three or more antibiotics simultaneously, as shown in Fig. 2.

The most frequent two-drug combination was ceftriaxone + metronidazole (56.5%), followed by ceftazidime + vancomycin (11.2%) and ceftriaxone + vancomycin (9%) (Table 5).

**Table 5:** Combinations of antibiotics prescribed for patients in the study wards, ACSH

Antibiotic combinations	Study Wards, *n* (%)				Total, *n* (%)
	Surgical	Medical	Pediatric	Gyn/Obs	
Ceftriaxone + azithromycin	0 (0)	30 (15.5)	2 (2.1)	0 (0)	32 (6.4)
Ceftriaxone + metronidazole	142 (88.2)	58 (29.9)	49 (52.7)	34 (64.1)	283 (56.5)
Ceftriaxone + vancomycin	0 (0)	36 (18.6)	8 (8.6)	1 (1.9)	45 (9.0)
Ceftriaxone + gentamicin	0 (0)	0 (0)	2 (2.1)	0 (0)	2 (0.4)
Metronidazole + vancomycin	2 (1.2)	4 (2.1)	2 (2.1)	0 (0)	8 (1.6)
Metronidazole + ciprofloxacin	0 (0)	4 (2.1)	1 (1.1)	0 (0)	5 (1.0)
Ceftazidime + vancomycin	10 (6.2)	28 (14.4)	18 (19.4)	0 (0)	56 (11.2)
Ciprofloxacin + vancomycin	1 (0.62)	1 (0.5)	3 (3.2)	0 (0)	5 (1.0)
Ceftriaxone + doxycycline	0 (0)	2 (1.0)	0 (0)	2 (3.8)	4 (0.8)
Cefazolin + ceftriaxone	0 (0)	0 (0)	0 (0)	5 (9.4)	5 (1.0)
Ceftazidime + metronidazole	4 (2.5)	6 (3.1)	0 (0)	0 (0)	10 (2.0)
Ceftriaxone + vancomycin + azithromycin	0 (0)	7 (3.6)	0 (0)	0 (0)	7 (1.4)
Ceftriaxone + vancomycin + metronidazole	2 (1.2)	6 (3.1)	3 (3.2)	0 (0)	11 (2.2)
Ceftazidime + vancomycin + azithromycin	0 (0)	2 (1.0)	4 (4.3)	0 (0)	6 (1.2)
Ceftriaxone + vancomycin + metronidazole	0 (0)	6 (3.1)	1 (1.1)	0 (0)	7 (1.4)
Ceftriaxone + metronidazole + ciprofloxacin	0 (0)	4 (2.1)	0 (0)	0 (0)	4 (0.8)
Ceftriaxone + metronidazole + doxycycline	0 (0)	0 (0)	0 (0)	3 (5.7)	3 (0.6)
Cefazolin + ceftriaxone + metronidazole	0 (0)	0 (0)	0 (0)	8 (15.1)	7 (1.4)
Total, n (%)	161(32.1)	194 (38.7)	93 (18.6)	53 (10.6)	501 (100)

#### Complementary indicators

**Fig. 3:**
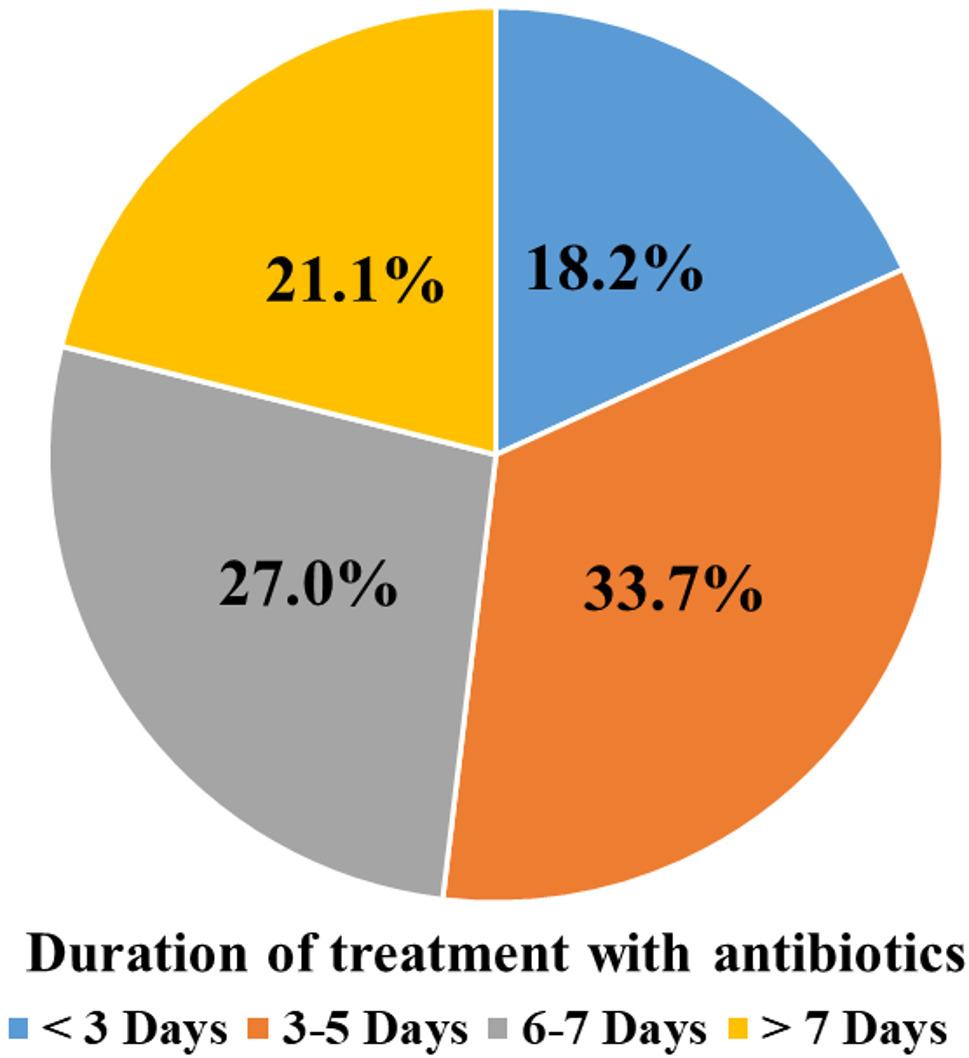
Duration of treatment with antibiotics prescribed for patients in the study wards, ACSH

Overall, patients received antibiotics for an average of 5.9 ± 3.9 days during their hospital stay; the longest individual course lasted 39 days in the surgical ward, reflecting a potential overuse of antibiotics in selected cases. When categorized by treatment length, the largest proportion of patients (33.7%) received antibiotics for 3–5 days, as shown in Fig. 3.

#### Factors associated with antibiotic use: generalized poisson regression (GPR)

Key predictors of the number of prescribed antibiotics included hospital stay, number of comorbidities, culture testing, therapeutic purpose, and specific conditions such as sepsis, pneumonia, stroke, pneumothorax, incomplete abortion, and other medical conditions (Table 6).

**Table 6:** GPR of socio-demographics and clinical factors with the number of antibiotics

Variable	IRR	Std. Err.	*p* value	95% CI	
Age	0.9991	0.0006	0.111	0.9980	1.0002
Sex*	0.9858	0.0071	0.046	0.9721	0.9998
Residence**	1.0624	0.0292	0.028	1.0066	1.1213
Number of days in hospital	1.0142	0.0019	0.013	1.0002	1.1026
Was pneumonia as a comorbid? ***	0.9997	0.0459	0.995	0.9137	1.0939
Was culture conducted? ***	1.4251	0.1101	0.000	1.2248	1.6580
Purpose of therapy****	0.9430	0.0286	0.032	0.8885	0.9894
Number of comorbid	1.0184	0.0026	0.000	1.0132	1.0236
Conditions*****					
Acute appendicitis	1.0184	0.0361	0.607	0.9501	1.0917
Abscess	1.1162	0.0929	0.186	0.9482	1.3140
Cholelithiasis	0.8826	0.0696	0.113	0.7562	1.0300
Parapneumonic Effusion/Empyema	1.3247	0.1263	0.003	1.0989	1.5968
Hemothorax	0.7858	0.0647	0.003	0.6688	0.9234
Incomplete abortion	0.3644	0.0155	0.000	0.3352	0.3962
Meningitis	1.1330	0.0734	0.054	0.9979	1.2863
Pyelonephritis	1.0359	0.0647	0.572	0.9165	1.1709
Opportunistic infections	1.2989	0.1042	0.001	1.1099	1.5200
Pneumonia	1.2431	0.0773	0.000	1.1004	1.4042
Peritonitis	1.0891	0.0737	0.207	0.9539	1.2435
Pneumothorax	0.6987	0.0636	0.000	0.5845	0.8351
Surgical prophylaxis	1				
Sepsis	1.5101	0.0886	0.000	1.3461	1.6940
Surgical site infections	1.6206	0.3166	0.013	1.1051	2.3765
Stroke	1.3456	0.0801	0.000	1.1975	1.5121
Traumatic injuries	0.9145	0.0304	0.007	0.8568	0.9761
Urinary tract infections	0.8241	0.0787	0.043	0.6834	0.9938
Other medical conditions	1.3047	0.0558	0.000	1.1999	1.4188
Constant	1.5657	0.1175	0.000	1.3516	1.8138

Dispersion (𝛂): − 0.8469; AIC: 1725.42; BIC: 1854.02

IRR: incidence rate ratio (IRR); AIC: Akaike’s information criterion; BIC: Bayesian information criterion; *: Male patients were the reference; **: those who came from urban areas were the reference; ***: Negative answer was the reference; ****: Therapeutic purpose was the reference; *****: Surgical prophylaxis was the reference

## Discussion

This study evaluated inpatient antibiotic prescribing patterns and antimicrobial stewardship (AMS) gaps using WHO antibiotic use indicators across major wards at Ayder Comprehensive Specialized Hospital (ACSH), Northern Ethiopia. Infectious diseases were the leading indications for antibiotic use, including acute appendicitis, pneumonia, pyelonephritis, and sepsis. Surgical prophylaxis also accounted for 24,5% of the prescriptions. Similar indications have been reported in other Ethiopian studies [24, 34]. While prophylaxis is essential for preventing surgical site infections, its high prevalence raises concerns regarding agent selection and duration, particularly in the absence of local guidelines [45, 46]. Over half of admitted patients (51.4%) received at least one antibiotic, which is consistent with findings from other Ethiopian referral hospitals [13, 14] and pooled African estimates, where inpatient antibiotic use typically ranges from 45% to 65% [19]. This confirms that high inpatient antibiotic exposure remains a persistent challenge in tertiary care settings in low- and middle-income countries (LMICs).

The mean number of antibiotics per patient (1.7) aligns with reports from Debre-Tabor, Ethiopia (Tadesse et al., 2022), but exceeds values reported in Eritrea [20], Palestine [21], Saudi Arabia [22], and India [23]. This difference likely reflects ACSH’s tertiary referral role, the management of more severe and complicated cases, widespread empirical prescribing, and inclusion of pediatric patients, where combination therapy is common.

Encouragingly, all prescribed antibiotics were listed in Ethiopia’s national Essential Medicines List (EML), and nearly all were prescribed by generic name. Similar findings have been reported from Axum [13] and Debre-Tabor [14] hospitals in Ethiopia, as well as from Eritrea [20] and Saudi Arabia [22]. These indicators reflect good adherence to national procurement and prescribing policies. However, this positive performance was overshadowed by the very high use of injectable antibiotics (90.9%), which exceeds earlier ACSH reports [17, 18] and findings from other Ethiopian hospitals [13, 14, 24] ranging from 78.7% to 82.4%. Excessive parenteral antibiotic use is concerning, given its association with higher costs, complications, and accelerated AMR [25, 26].

More than half of antibiotic use was for therapeutic purposes, and nearly all therapeutic prescriptions were empirical, consistent with findings from Axum, Ethiopia [13]. Culture and antimicrobial susceptibility testing were rarely performed, despite ACSH being a tertiary referral center. Similar underutilization of microbiological services has been reported in other LMIC settings and represents a major stewardship gap [27–29]. Logistical challenges, including inconsistent laboratory supplies, likely contribute to this practice, reinforcing reliance on empirical treatment [30].

Ceftriaxone was the most frequently prescribed antibiotic, followed by metronidazole and vancomycin, with increased use compared to earlier ACSH studies [17, 18]. The magnitude of ceftriaxone use observed here exceeds several reports from Ethiopia [13], Eritrea [20] and Sierra Leone [31]. While ceftriaxone’s broad-spectrum activity supports its empirical use, studies from Ethiopia have documented high levels of inappropriate prescribing, raising concerns regarding resistance selection [32–35].

Consistent with this pattern, most prescribed antibiotics belonged to the WHO Watch category, exceeding the recommendation that at least 70% of antibiotic use should come from the Access group [36]. Similar overuse of Watch antibiotics has been reported in several LMICs and is often driven by prescriber preference, fear of treatment failure, and drug availability [37–40].

Combination therapy was common, particularly ceftriaxone combined with metronidazole, reflecting prevailing practices in surgical and intra-abdominal infections. While such combinations may be clinically justified in selected cases, their widespread use reveals the need for clearer institutional guidelines and regular prescription review ([41, 42].

The mean duration of antibiotic therapy was approximately six days, with one-third of patients receiving treatment for more than seven days. Comparable durations have been reported in Debre-Tabor, Ethiopia [14] and Eritrea [20] but were longer than those reported in Axum, Ethiopia [13] and India [23]. Although, the study included heterogeneous indications that may require variable treatment duration, treatment duration using institutional guidelines is crucial for mitigating resistance and is among the key issues of stewardship [43, 44].

Regression analysis identified several factors associated with increased antibiotic use, including rural residence, longer hospital stays, comorbidities, and severe clinical conditions such as sepsis and pneumonia. These findings are consistent with evidence from multiple settings indicating increased antibiotic exposure among patients with greater disease severity and complexity [45–51]. In contrast, prophylactic prescriptions and female sex were associated with fewer antibiotics, likely reflecting standardized prophylaxis practices in cesarean section cases [50].

Overall, this study demonstrates persistent AMS gaps at ACSH, characterized by high empirical prescribing, excessive use of injectable and Watch-category antibiotics, limited microbiological testing, and reliance on broad-spectrum agents. Strengthening diagnostic capacity, developing local treatment guidelines, and implementing targeted AMS interventions are critical to improving rational antibiotic use and mitigating AMR.

### Limitations of the study

This study has several limitations. It was conducted in a single tertiary hospital in Northern Ethiopia, which may limit the generalizability of the findings to other healthcare settings. In addition, the study focused exclusively on inpatients; therefore, antibiotic prescribing practices in outpatient settings were not assessed. Despite these limitations, the study provides important insights into inpatient antibiotic use and AMS gaps in a major referral hospital. Additionally, the regression analysis did not adjust for ward-level clustering, and residual confounding by ward cannot be excluded.

## Conclusion

This study assessed inpatient antibiotic prescribing patterns at Ayder Comprehensive Specialized Hospital (ACSH) using WHO antibiotic use indicators. More than half of hospitalized patients received at least one antibiotic, predominantly for empirical therapy, despite full adherence to the national Essential Medicines List. Limited use of culture and antimicrobial susceptibility testing, overuse of Watch-category antibiotics, high reliance on injectable formulations, and the absence of standardized treatment guidelines reveal critical AMS gaps. Longer hospital stays, comorbidities, and severe clinical conditions, particularly sepsis, pneumonia, and stroke, were key determinants of increased antibiotic use. Strengthening AMS through hospital-specific treatment guidelines, improved diagnostic capacity, and continuous prescriber training is essential to promote rational antibiotic use and slow the occurrence AMR in Ethiopian hospitals.

## Data Availability

The data used and/or analyzed during the study are available from the corresponding author upon reasonable request.

## Abbreviations

ACSH: Ayder Comprehensive Specialized Hospital
AMR: Antimicrobial resistance
EML: Essential medicine list
AMS: Antimicrobial stewardship
GPR: Generalized poisson regression
Gyn/Obs: Gynecology/obstetrics
LMICs: Low- and Middle-Income Countries
WHO: World Health Organization
